# Supplementary material for: Optimal minimal residual disease threshold in pediatric acute myeloid leukemia: A retrospective cohort study based on the TARGET database
Source: PLoS Med. 2026 May 8;23(5):e1005088. doi: 10.1371/journal.pmed.1005088 (PMC13155632; doi:10.1371/journal.pmed.1005088)
Supplement: S1 Code — (ZIP) [file pmed.1005088.s002.zip › S2 code/PROJ8_11_tbl/PROJ8_11_tbl.htm]

## Kaplan Meier Survival Curve

Outcome: First Event
Time: EFS(years)
Landmark analysis for time segment: EFS(years) <= 5
Log rank test: implements the G-rho family of Harrington and Fleming (1982), with weights on each death of S(t)^rho, where S is the Kaplan-Meier estimate of survival. With rho = 0 this is the log-rank or Mantel-Haenszel test.

|  |  |  |  |
| --- | --- | --- | --- |
|  | N | Observed | Expected |
| factor(X25.CS)=0 | 490 | 297 | 341.1979 |
| factor(X25.CS)=1 | 84 | 74 | 29.8021 |

Chisq=
72.1686
on
1
degree of freedom, p=
<0.0001
Survival table

|  |  |  |  |  |  |  |  |
| --- | --- | --- | --- | --- | --- | --- | --- |
|  | EFS(years) | N.Risk | N.Event | N.Censor | Survival | 95%CI Low | 95%CI Upp |
| factor(X25.CS)=0 | 0.16 | 490 | 1 | 0 | 0.9980 | 0.9940 | 1.0000 |
| factor(X25.CS)=0 | 0.17 | 489 | 2 | 0 | 0.9939 | 0.9870 | 1.0000 |
| factor(X25.CS)=0 | 0.18 | 487 | 3 | 0 | 0.9878 | 0.9781 | 0.9975 |
| factor(X25.CS)=0 | 0.19 | 484 | 0 | 1 | 0.9878 | 0.9781 | 0.9975 |
| factor(X25.CS)=0 | 0.2 | 483 | 5 | 0 | 0.9775 | 0.9645 | 0.9907 |
| factor(X25.CS)=0 | 0.21 | 478 | 3 | 0 | 0.9714 | 0.9567 | 0.9863 |
| factor(X25.CS)=0 | 0.23 | 475 | 0 | 1 | 0.9714 | 0.9567 | 0.9863 |
| factor(X25.CS)=0 | 0.24 | 474 | 1 | 0 | 0.9693 | 0.9542 | 0.9847 |
| factor(X25.CS)=0 | 0.25 | 473 | 1 | 0 | 0.9673 | 0.9517 | 0.9832 |
| factor(X25.CS)=0 | 0.26 | 472 | 2 | 0 | 0.9632 | 0.9467 | 0.9800 |
| factor(X25.CS)=0 | 0.27 | 470 | 3 | 1 | 0.9570 | 0.9392 | 0.9752 |
| factor(X25.CS)=0 | 0.28 | 466 | 4 | 0 | 0.9488 | 0.9295 | 0.9686 |
| factor(X25.CS)=0 | 0.29 | 462 | 1 | 0 | 0.9468 | 0.9271 | 0.9669 |
| factor(X25.CS)=0 | 0.31 | 461 | 1 | 0 | 0.9447 | 0.9247 | 0.9652 |
| factor(X25.CS)=0 | 0.32 | 460 | 1 | 0 | 0.9427 | 0.9223 | 0.9635 |
| factor(X25.CS)=0 | 0.33 | 459 | 2 | 1 | 0.9386 | 0.9175 | 0.9601 |
| factor(X25.CS)=0 | 0.35 | 456 | 1 | 0 | 0.9365 | 0.9151 | 0.9584 |
| factor(X25.CS)=0 | 0.36 | 455 | 1 | 0 | 0.9344 | 0.9127 | 0.9567 |
| factor(X25.CS)=0 | 0.37 | 454 | 2 | 0 | 0.9303 | 0.9080 | 0.9532 |
| factor(X25.CS)=0 | 0.38 | 452 | 2 | 0 | 0.9262 | 0.9033 | 0.9497 |
| factor(X25.CS)=0 | 0.4 | 450 | 4 | 0 | 0.9180 | 0.8939 | 0.9427 |
| factor(X25.CS)=0 | 0.41 | 446 | 1 | 0 | 0.9159 | 0.8916 | 0.9409 |
| factor(X25.CS)=0 | 0.42 | 445 | 1 | 0 | 0.9139 | 0.8893 | 0.9391 |
| factor(X25.CS)=0 | 0.43 | 444 | 1 | 0 | 0.9118 | 0.8870 | 0.9373 |
| factor(X25.CS)=0 | 0.44 | 443 | 3 | 0 | 0.9056 | 0.8800 | 0.9320 |
| factor(X25.CS)=0 | 0.45 | 440 | 2 | 0 | 0.9015 | 0.8754 | 0.9284 |
| factor(X25.CS)=0 | 0.46 | 438 | 2 | 0 | 0.8974 | 0.8709 | 0.9248 |
| factor(X25.CS)=0 | 0.47 | 436 | 2 | 0 | 0.8933 | 0.8663 | 0.9211 |
| factor(X25.CS)=0 | 0.48 | 434 | 2 | 0 | 0.8892 | 0.8617 | 0.9175 |
| factor(X25.CS)=0 | 0.5 | 432 | 2 | 0 | 0.8851 | 0.8572 | 0.9138 |
| factor(X25.CS)=0 | 0.51 | 430 | 1 | 0 | 0.8830 | 0.8549 | 0.9120 |
| factor(X25.CS)=0 | 0.52 | 429 | 4 | 0 | 0.8748 | 0.8458 | 0.9047 |
| factor(X25.CS)=0 | 0.53 | 425 | 2 | 0 | 0.8706 | 0.8413 | 0.9010 |
| factor(X25.CS)=0 | 0.54 | 423 | 3 | 0 | 0.8645 | 0.8346 | 0.8954 |
| factor(X25.CS)=0 | 0.55 | 420 | 3 | 0 | 0.8583 | 0.8279 | 0.8898 |
| factor(X25.CS)=0 | 0.56 | 417 | 7 | 0 | 0.8439 | 0.8122 | 0.8768 |
| factor(X25.CS)=0 | 0.57 | 410 | 2 | 0 | 0.8398 | 0.8078 | 0.8730 |
| factor(X25.CS)=0 | 0.58 | 408 | 4 | 0 | 0.8315 | 0.7989 | 0.8655 |
| factor(X25.CS)=0 | 0.59 | 404 | 4 | 0 | 0.8233 | 0.7901 | 0.8579 |
| factor(X25.CS)=0 | 0.6 | 400 | 3 | 0 | 0.8171 | 0.7835 | 0.8522 |
| factor(X25.CS)=0 | 0.62 | 397 | 2 | 0 | 0.8130 | 0.7791 | 0.8484 |
| factor(X25.CS)=0 | 0.63 | 395 | 1 | 0 | 0.8110 | 0.7769 | 0.8465 |
| factor(X25.CS)=0 | 0.64 | 394 | 1 | 0 | 0.8089 | 0.7747 | 0.8446 |
| factor(X25.CS)=0 | 0.65 | 393 | 4 | 0 | 0.8007 | 0.7659 | 0.8370 |
| factor(X25.CS)=0 | 0.67 | 389 | 1 | 0 | 0.7986 | 0.7637 | 0.8350 |
| factor(X25.CS)=0 | 0.68 | 388 | 5 | 0 | 0.7883 | 0.7528 | 0.8255 |
| factor(X25.CS)=0 | 0.69 | 383 | 5 | 0 | 0.7780 | 0.7420 | 0.8158 |
| factor(X25.CS)=0 | 0.7 | 378 | 4 | 0 | 0.7698 | 0.7333 | 0.8081 |
| factor(X25.CS)=0 | 0.71 | 374 | 2 | 0 | 0.7657 | 0.7289 | 0.8043 |
| factor(X25.CS)=0 | 0.72 | 372 | 5 | 0 | 0.7554 | 0.7181 | 0.7946 |
| factor(X25.CS)=0 | 0.73 | 367 | 4 | 0 | 0.7471 | 0.7095 | 0.7868 |
| factor(X25.CS)=0 | 0.74 | 363 | 2 | 0 | 0.7430 | 0.7052 | 0.7829 |
| factor(X25.CS)=0 | 0.75 | 361 | 2 | 0 | 0.7389 | 0.7009 | 0.7790 |
| factor(X25.CS)=0 | 0.76 | 359 | 7 | 0 | 0.7245 | 0.6859 | 0.7653 |
| factor(X25.CS)=0 | 0.77 | 352 | 1 | 0 | 0.7224 | 0.6837 | 0.7634 |
| factor(X25.CS)=0 | 0.78 | 351 | 3 | 1 | 0.7163 | 0.6773 | 0.7575 |
| factor(X25.CS)=0 | 0.79 | 347 | 4 | 0 | 0.7080 | 0.6687 | 0.7496 |
| factor(X25.CS)=0 | 0.8 | 343 | 3 | 0 | 0.7018 | 0.6623 | 0.7437 |
| factor(X25.CS)=0 | 0.81 | 340 | 5 | 0 | 0.6915 | 0.6516 | 0.7338 |
| factor(X25.CS)=0 | 0.82 | 335 | 4 | 0 | 0.6832 | 0.6431 | 0.7259 |
| factor(X25.CS)=0 | 0.83 | 331 | 4 | 0 | 0.6750 | 0.6346 | 0.7179 |
| factor(X25.CS)=0 | 0.84 | 327 | 2 | 0 | 0.6709 | 0.6304 | 0.7140 |
| factor(X25.CS)=0 | 0.86 | 325 | 1 | 0 | 0.6688 | 0.6282 | 0.7120 |
| factor(X25.CS)=0 | 0.88 | 324 | 4 | 0 | 0.6605 | 0.6197 | 0.7040 |
| factor(X25.CS)=0 | 0.89 | 320 | 1 | 0 | 0.6585 | 0.6176 | 0.7020 |
| factor(X25.CS)=0 | 0.9 | 319 | 2 | 0 | 0.6543 | 0.6134 | 0.6980 |
| factor(X25.CS)=0 | 0.91 | 317 | 1 | 0 | 0.6523 | 0.6113 | 0.6960 |
| factor(X25.CS)=0 | 0.92 | 316 | 5 | 0 | 0.6420 | 0.6007 | 0.6860 |
| factor(X25.CS)=0 | 0.93 | 311 | 6 | 0 | 0.6296 | 0.5881 | 0.6740 |
| factor(X25.CS)=0 | 0.95 | 305 | 1 | 0 | 0.6275 | 0.5860 | 0.6720 |
| factor(X25.CS)=0 | 0.96 | 304 | 0 | 1 | 0.6275 | 0.5860 | 0.6720 |
| factor(X25.CS)=0 | 0.97 | 303 | 1 | 0 | 0.6254 | 0.5838 | 0.6700 |
| factor(X25.CS)=0 | 0.98 | 302 | 1 | 0 | 0.6234 | 0.5817 | 0.6680 |
| factor(X25.CS)=0 | 0.99 | 301 | 3 | 0 | 0.6172 | 0.5754 | 0.6619 |
| factor(X25.CS)=0 | 1 | 298 | 3 | 0 | 0.6109 | 0.5691 | 0.6559 |
| factor(X25.CS)=0 | 1.01 | 295 | 2 | 0 | 0.6068 | 0.5649 | 0.6519 |
| factor(X25.CS)=0 | 1.02 | 293 | 2 | 0 | 0.6027 | 0.5607 | 0.6478 |
| factor(X25.CS)=0 | 1.03 | 291 | 2 | 0 | 0.5985 | 0.5565 | 0.6438 |
| factor(X25.CS)=0 | 1.05 | 289 | 2 | 0 | 0.5944 | 0.5523 | 0.6397 |
| factor(X25.CS)=0 | 1.06 | 287 | 1 | 0 | 0.5923 | 0.5502 | 0.6377 |
| factor(X25.CS)=0 | 1.07 | 286 | 3 | 0 | 0.5861 | 0.5439 | 0.6316 |
| factor(X25.CS)=0 | 1.08 | 283 | 4 | 0 | 0.5778 | 0.5355 | 0.6235 |
| factor(X25.CS)=0 | 1.1 | 279 | 1 | 0 | 0.5757 | 0.5334 | 0.6214 |
| factor(X25.CS)=0 | 1.12 | 278 | 2 | 0 | 0.5716 | 0.5292 | 0.6174 |
| factor(X25.CS)=0 | 1.13 | 276 | 1 | 0 | 0.5695 | 0.5271 | 0.6153 |
| factor(X25.CS)=0 | 1.14 | 275 | 2 | 0 | 0.5654 | 0.5229 | 0.6113 |
| factor(X25.CS)=0 | 1.18 | 273 | 1 | 0 | 0.5633 | 0.5209 | 0.6092 |
| factor(X25.CS)=0 | 1.19 | 272 | 2 | 0 | 0.5592 | 0.5167 | 0.6052 |
| factor(X25.CS)=0 | 1.2 | 270 | 3 | 0 | 0.5530 | 0.5104 | 0.5990 |
| factor(X25.CS)=0 | 1.21 | 267 | 0 | 1 | 0.5530 | 0.5104 | 0.5990 |
| factor(X25.CS)=0 | 1.25 | 266 | 2 | 0 | 0.5488 | 0.5062 | 0.5949 |
| factor(X25.CS)=0 | 1.26 | 264 | 1 | 0 | 0.5467 | 0.5042 | 0.5929 |
| factor(X25.CS)=0 | 1.27 | 263 | 2 | 0 | 0.5426 | 0.5000 | 0.5888 |
| factor(X25.CS)=0 | 1.28 | 261 | 1 | 0 | 0.5405 | 0.4979 | 0.5867 |
| factor(X25.CS)=0 | 1.3 | 260 | 3 | 0 | 0.5342 | 0.4916 | 0.5806 |
| factor(X25.CS)=0 | 1.31 | 257 | 2 | 0 | 0.5301 | 0.4875 | 0.5764 |
| factor(X25.CS)=0 | 1.34 | 255 | 3 | 0 | 0.5239 | 0.4812 | 0.5703 |
| factor(X25.CS)=0 | 1.35 | 252 | 2 | 0 | 0.5197 | 0.4771 | 0.5661 |
| factor(X25.CS)=0 | 1.36 | 250 | 2 | 0 | 0.5155 | 0.4729 | 0.5620 |
| factor(X25.CS)=0 | 1.37 | 248 | 1 | 0 | 0.5135 | 0.4708 | 0.5600 |
| factor(X25.CS)=0 | 1.38 | 247 | 3 | 0 | 0.5072 | 0.4646 | 0.5538 |
| factor(X25.CS)=0 | 1.39 | 244 | 2 | 0 | 0.5031 | 0.4604 | 0.5496 |
| factor(X25.CS)=0 | 1.4 | 242 | 1 | 0 | 0.5010 | 0.4584 | 0.5476 |
| factor(X25.CS)=0 | 1.41 | 241 | 1 | 0 | 0.4989 | 0.4563 | 0.5455 |
| factor(X25.CS)=0 | 1.42 | 240 | 1 | 0 | 0.4968 | 0.4542 | 0.5434 |
| factor(X25.CS)=0 | 1.43 | 239 | 3 | 0 | 0.4906 | 0.4480 | 0.5372 |
| factor(X25.CS)=0 | 1.45 | 236 | 1 | 0 | 0.4885 | 0.4460 | 0.5351 |
| factor(X25.CS)=0 | 1.46 | 235 | 1 | 0 | 0.4864 | 0.4439 | 0.5331 |
| factor(X25.CS)=0 | 1.49 | 234 | 1 | 0 | 0.4844 | 0.4418 | 0.5310 |
| factor(X25.CS)=0 | 1.5 | 233 | 1 | 0 | 0.4823 | 0.4398 | 0.5289 |
| factor(X25.CS)=0 | 1.51 | 232 | 1 | 0 | 0.4802 | 0.4377 | 0.5268 |
| factor(X25.CS)=0 | 1.53 | 231 | 1 | 0 | 0.4781 | 0.4356 | 0.5248 |
| factor(X25.CS)=0 | 1.56 | 230 | 1 | 0 | 0.4760 | 0.4336 | 0.5227 |
| factor(X25.CS)=0 | 1.58 | 229 | 4 | 0 | 0.4677 | 0.4253 | 0.5144 |
| factor(X25.CS)=0 | 1.6 | 225 | 2 | 0 | 0.4636 | 0.4212 | 0.5102 |
| factor(X25.CS)=0 | 1.62 | 223 | 2 | 0 | 0.4594 | 0.4171 | 0.5060 |
| factor(X25.CS)=0 | 1.64 | 221 | 1 | 0 | 0.4573 | 0.4150 | 0.5040 |
| factor(X25.CS)=0 | 1.72 | 220 | 2 | 0 | 0.4532 | 0.4109 | 0.4998 |
| factor(X25.CS)=0 | 1.75 | 218 | 0 | 1 | 0.4532 | 0.4109 | 0.4998 |
| factor(X25.CS)=0 | 1.77 | 217 | 1 | 0 | 0.4511 | 0.4088 | 0.4977 |
| factor(X25.CS)=0 | 1.89 | 216 | 2 | 0 | 0.4469 | 0.4047 | 0.4935 |
| factor(X25.CS)=0 | 1.9 | 214 | 2 | 0 | 0.4427 | 0.4006 | 0.4893 |
| factor(X25.CS)=0 | 1.92 | 212 | 1 | 0 | 0.4406 | 0.3985 | 0.4872 |
| factor(X25.CS)=0 | 1.98 | 211 | 4 | 0 | 0.4323 | 0.3903 | 0.4788 |
| factor(X25.CS)=0 | 2.05 | 207 | 1 | 0 | 0.4302 | 0.3882 | 0.4767 |
| factor(X25.CS)=0 | 2.07 | 206 | 1 | 0 | 0.4281 | 0.3862 | 0.4746 |
| factor(X25.CS)=0 | 2.08 | 205 | 1 | 0 | 0.4260 | 0.3841 | 0.4725 |
| factor(X25.CS)=0 | 2.11 | 204 | 1 | 0 | 0.4239 | 0.3821 | 0.4704 |
| factor(X25.CS)=0 | 2.13 | 203 | 1 | 0 | 0.4219 | 0.3800 | 0.4683 |
| factor(X25.CS)=0 | 2.15 | 202 | 1 | 0 | 0.4198 | 0.3780 | 0.4662 |
| factor(X25.CS)=0 | 2.18 | 201 | 0 | 1 | 0.4198 | 0.3780 | 0.4662 |
| factor(X25.CS)=0 | 2.2 | 200 | 1 | 0 | 0.4177 | 0.3759 | 0.4640 |
| factor(X25.CS)=0 | 2.22 | 199 | 2 | 0 | 0.4135 | 0.3718 | 0.4598 |
| factor(X25.CS)=0 | 2.23 | 197 | 0 | 1 | 0.4135 | 0.3718 | 0.4598 |
| factor(X25.CS)=0 | 2.34 | 196 | 1 | 0 | 0.4114 | 0.3697 | 0.4577 |
| factor(X25.CS)=0 | 2.36 | 195 | 1 | 0 | 0.4092 | 0.3677 | 0.4555 |
| factor(X25.CS)=0 | 2.43 | 194 | 0 | 1 | 0.4092 | 0.3677 | 0.4555 |
| factor(X25.CS)=0 | 2.49 | 193 | 0 | 1 | 0.4092 | 0.3677 | 0.4555 |
| factor(X25.CS)=0 | 2.5 | 192 | 0 | 1 | 0.4092 | 0.3677 | 0.4555 |
| factor(X25.CS)=0 | 2.51 | 191 | 0 | 1 | 0.4092 | 0.3677 | 0.4555 |
| factor(X25.CS)=0 | 2.53 | 190 | 1 | 0 | 0.4071 | 0.3655 | 0.4534 |
| factor(X25.CS)=0 | 2.56 | 189 | 0 | 1 | 0.4071 | 0.3655 | 0.4534 |
| factor(X25.CS)=0 | 2.61 | 188 | 1 | 1 | 0.4049 | 0.3634 | 0.4512 |
| factor(X25.CS)=0 | 2.62 | 186 | 2 | 0 | 0.4006 | 0.3591 | 0.4468 |
| factor(X25.CS)=0 | 2.63 | 184 | 0 | 1 | 0.4006 | 0.3591 | 0.4468 |
| factor(X25.CS)=0 | 2.71 | 183 | 0 | 1 | 0.4006 | 0.3591 | 0.4468 |
| factor(X25.CS)=0 | 2.83 | 182 | 1 | 0 | 0.3984 | 0.3570 | 0.4446 |
| factor(X25.CS)=0 | 2.89 | 181 | 0 | 1 | 0.3984 | 0.3570 | 0.4446 |
| factor(X25.CS)=0 | 2.98 | 180 | 0 | 1 | 0.3984 | 0.3570 | 0.4446 |
| factor(X25.CS)=0 | 3.05 | 179 | 0 | 2 | 0.3984 | 0.3570 | 0.4446 |
| factor(X25.CS)=0 | 3.07 | 177 | 0 | 1 | 0.3984 | 0.3570 | 0.4446 |
| factor(X25.CS)=0 | 3.16 | 176 | 0 | 1 | 0.3984 | 0.3570 | 0.4446 |
| factor(X25.CS)=0 | 3.18 | 175 | 1 | 3 | 0.3961 | 0.3547 | 0.4423 |
| factor(X25.CS)=0 | 3.25 | 171 | 0 | 1 | 0.3961 | 0.3547 | 0.4423 |
| factor(X25.CS)=0 | 3.28 | 170 | 0 | 1 | 0.3961 | 0.3547 | 0.4423 |
| factor(X25.CS)=0 | 3.29 | 169 | 0 | 1 | 0.3961 | 0.3547 | 0.4423 |
| factor(X25.CS)=0 | 3.3 | 168 | 0 | 1 | 0.3961 | 0.3547 | 0.4423 |
| factor(X25.CS)=0 | 3.33 | 167 | 0 | 1 | 0.3961 | 0.3547 | 0.4423 |
| factor(X25.CS)=0 | 3.35 | 166 | 0 | 2 | 0.3961 | 0.3547 | 0.4423 |
| factor(X25.CS)=0 | 3.36 | 164 | 0 | 1 | 0.3961 | 0.3547 | 0.4423 |
| factor(X25.CS)=0 | 3.38 | 163 | 0 | 2 | 0.3961 | 0.3547 | 0.4423 |
| factor(X25.CS)=0 | 3.39 | 161 | 1 | 1 | 0.3936 | 0.3523 | 0.4398 |
| factor(X25.CS)=0 | 3.4 | 159 | 0 | 1 | 0.3936 | 0.3523 | 0.4398 |
| factor(X25.CS)=0 | 3.41 | 158 | 0 | 3 | 0.3936 | 0.3523 | 0.4398 |
| factor(X25.CS)=0 | 3.44 | 155 | 0 | 2 | 0.3936 | 0.3523 | 0.4398 |
| factor(X25.CS)=0 | 3.45 | 153 | 0 | 1 | 0.3936 | 0.3523 | 0.4398 |
| factor(X25.CS)=0 | 3.46 | 152 | 0 | 1 | 0.3936 | 0.3523 | 0.4398 |
| factor(X25.CS)=0 | 3.48 | 151 | 0 | 1 | 0.3936 | 0.3523 | 0.4398 |
| factor(X25.CS)=0 | 3.5 | 150 | 0 | 1 | 0.3936 | 0.3523 | 0.4398 |
| factor(X25.CS)=0 | 3.53 | 149 | 0 | 1 | 0.3936 | 0.3523 | 0.4398 |
| factor(X25.CS)=0 | 3.56 | 148 | 0 | 1 | 0.3936 | 0.3523 | 0.4398 |
| factor(X25.CS)=0 | 3.62 | 147 | 0 | 1 | 0.3936 | 0.3523 | 0.4398 |
| factor(X25.CS)=0 | 3.63 | 146 | 0 | 1 | 0.3936 | 0.3523 | 0.4398 |
| factor(X25.CS)=0 | 3.81 | 145 | 0 | 1 | 0.3936 | 0.3523 | 0.4398 |
| factor(X25.CS)=0 | 3.84 | 144 | 0 | 1 | 0.3936 | 0.3523 | 0.4398 |
| factor(X25.CS)=0 | 3.88 | 143 | 0 | 1 | 0.3936 | 0.3523 | 0.4398 |
| factor(X25.CS)=0 | 3.96 | 142 | 0 | 1 | 0.3936 | 0.3523 | 0.4398 |
| factor(X25.CS)=0 | 3.99 | 141 | 1 | 0 | 0.3908 | 0.3495 | 0.4371 |
| factor(X25.CS)=0 | 4.04 | 140 | 0 | 2 | 0.3908 | 0.3495 | 0.4371 |
| factor(X25.CS)=0 | 4.07 | 138 | 1 | 0 | 0.3880 | 0.3466 | 0.4343 |
| factor(X25.CS)=0 | 4.08 | 137 | 0 | 1 | 0.3880 | 0.3466 | 0.4343 |
| factor(X25.CS)=0 | 4.19 | 136 | 0 | 2 | 0.3880 | 0.3466 | 0.4343 |
| factor(X25.CS)=0 | 4.22 | 134 | 0 | 2 | 0.3880 | 0.3466 | 0.4343 |
| factor(X25.CS)=0 | 4.23 | 132 | 1 | 0 | 0.3851 | 0.3437 | 0.4315 |
| factor(X25.CS)=0 | 4.27 | 131 | 0 | 1 | 0.3851 | 0.3437 | 0.4315 |
| factor(X25.CS)=0 | 4.31 | 130 | 0 | 1 | 0.3851 | 0.3437 | 0.4315 |
| factor(X25.CS)=0 | 4.34 | 129 | 0 | 1 | 0.3851 | 0.3437 | 0.4315 |
| factor(X25.CS)=0 | 4.35 | 128 | 0 | 1 | 0.3851 | 0.3437 | 0.4315 |
| factor(X25.CS)=0 | 4.38 | 127 | 0 | 2 | 0.3851 | 0.3437 | 0.4315 |
| factor(X25.CS)=0 | 4.41 | 125 | 0 | 1 | 0.3851 | 0.3437 | 0.4315 |
| factor(X25.CS)=0 | 4.42 | 124 | 0 | 1 | 0.3851 | 0.3437 | 0.4315 |
| factor(X25.CS)=0 | 4.44 | 123 | 0 | 2 | 0.3851 | 0.3437 | 0.4315 |
| factor(X25.CS)=0 | 4.45 | 121 | 0 | 1 | 0.3851 | 0.3437 | 0.4315 |
| factor(X25.CS)=0 | 4.47 | 120 | 0 | 1 | 0.3851 | 0.3437 | 0.4315 |
| factor(X25.CS)=0 | 4.48 | 119 | 0 | 1 | 0.3851 | 0.3437 | 0.4315 |
| factor(X25.CS)=0 | 4.49 | 118 | 0 | 2 | 0.3851 | 0.3437 | 0.4315 |
| factor(X25.CS)=0 | 4.5 | 116 | 0 | 1 | 0.3851 | 0.3437 | 0.4315 |
| factor(X25.CS)=0 | 4.53 | 115 | 0 | 3 | 0.3851 | 0.3437 | 0.4315 |
| factor(X25.CS)=0 | 4.55 | 112 | 0 | 1 | 0.3851 | 0.3437 | 0.4315 |
| factor(X25.CS)=0 | 4.58 | 111 | 0 | 2 | 0.3851 | 0.3437 | 0.4315 |
| factor(X25.CS)=0 | 4.6 | 109 | 0 | 2 | 0.3851 | 0.3437 | 0.4315 |
| factor(X25.CS)=0 | 4.62 | 107 | 0 | 1 | 0.3851 | 0.3437 | 0.4315 |
| factor(X25.CS)=0 | 4.65 | 106 | 0 | 1 | 0.3851 | 0.3437 | 0.4315 |
| factor(X25.CS)=0 | 4.7 | 105 | 0 | 1 | 0.3851 | 0.3437 | 0.4315 |
| factor(X25.CS)=0 | 4.72 | 104 | 1 | 0 | 0.3814 | 0.3398 | 0.4280 |
| factor(X25.CS)=0 | 4.79 | 103 | 0 | 1 | 0.3814 | 0.3398 | 0.4280 |
| factor(X25.CS)=0 | 4.81 | 102 | 0 | 1 | 0.3814 | 0.3398 | 0.4280 |
| factor(X25.CS)=0 | 4.85 | 101 | 0 | 3 | 0.3814 | 0.3398 | 0.4280 |
| factor(X25.CS)=0 | 4.88 | 98 | 0 | 1 | 0.3814 | 0.3398 | 0.4280 |
| factor(X25.CS)=0 | 4.9 | 97 | 0 | 1 | 0.3814 | 0.3398 | 0.4280 |
| factor(X25.CS)=0 | 4.99 | 96 | 0 | 1 | 0.3814 | 0.3398 | 0.4280 |
| factor(X25.CS)=0 | 5 | 95 | 0 | 95 | 0.3814 | 0.3398 | 0.4280 |
| factor(X25.CS)=1 | 0.15 | 84 | 1 | 0 | 0.9881 | 0.9652 | 1.0000 |
| factor(X25.CS)=1 | 0.16 | 83 | 3 | 0 | 0.9524 | 0.9079 | 0.9990 |
| factor(X25.CS)=1 | 0.17 | 80 | 2 | 0 | 0.9286 | 0.8751 | 0.9853 |
| factor(X25.CS)=1 | 0.19 | 78 | 7 | 0 | 0.8452 | 0.7713 | 0.9262 |
| factor(X25.CS)=1 | 0.2 | 71 | 2 | 0 | 0.8214 | 0.7435 | 0.9076 |
| factor(X25.CS)=1 | 0.21 | 69 | 9 | 0 | 0.7143 | 0.6239 | 0.8177 |
| factor(X25.CS)=1 | 0.22 | 60 | 7 | 0 | 0.6310 | 0.5358 | 0.7431 |
| factor(X25.CS)=1 | 0.23 | 53 | 7 | 0 | 0.5476 | 0.4509 | 0.6651 |
| factor(X25.CS)=1 | 0.24 | 46 | 1 | 0 | 0.5357 | 0.4390 | 0.6537 |
| factor(X25.CS)=1 | 0.25 | 45 | 1 | 0 | 0.5238 | 0.4272 | 0.6423 |
| factor(X25.CS)=1 | 0.27 | 44 | 1 | 0 | 0.5119 | 0.4154 | 0.6308 |
| factor(X25.CS)=1 | 0.28 | 43 | 1 | 0 | 0.5000 | 0.4037 | 0.6192 |
| factor(X25.CS)=1 | 0.3 | 42 | 3 | 0 | 0.4643 | 0.3690 | 0.5842 |
| factor(X25.CS)=1 | 0.32 | 39 | 1 | 0 | 0.4524 | 0.3575 | 0.5724 |
| factor(X25.CS)=1 | 0.35 | 38 | 1 | 0 | 0.4405 | 0.3461 | 0.5605 |
| factor(X25.CS)=1 | 0.49 | 37 | 2 | 0 | 0.4167 | 0.3235 | 0.5366 |
| factor(X25.CS)=1 | 0.53 | 35 | 1 | 0 | 0.4048 | 0.3123 | 0.5246 |
| factor(X25.CS)=1 | 0.59 | 34 | 1 | 0 | 0.3929 | 0.3011 | 0.5125 |
| factor(X25.CS)=1 | 0.64 | 33 | 1 | 0 | 0.3810 | 0.2901 | 0.5003 |
| factor(X25.CS)=1 | 0.69 | 32 | 1 | 0 | 0.3690 | 0.2790 | 0.4881 |
| factor(X25.CS)=1 | 0.7 | 31 | 2 | 0 | 0.3452 | 0.2572 | 0.4635 |
| factor(X25.CS)=1 | 0.74 | 29 | 1 | 0 | 0.3333 | 0.2463 | 0.4510 |
| factor(X25.CS)=1 | 0.77 | 28 | 2 | 0 | 0.3095 | 0.2249 | 0.4260 |
| factor(X25.CS)=1 | 0.78 | 26 | 1 | 0 | 0.2976 | 0.2143 | 0.4134 |
| factor(X25.CS)=1 | 0.87 | 25 | 1 | 0 | 0.2857 | 0.2037 | 0.4007 |
| factor(X25.CS)=1 | 0.9 | 24 | 1 | 0 | 0.2738 | 0.1933 | 0.3879 |
| factor(X25.CS)=1 | 0.95 | 23 | 1 | 0 | 0.2619 | 0.1829 | 0.3750 |
| factor(X25.CS)=1 | 1.02 | 22 | 1 | 0 | 0.2500 | 0.1726 | 0.3621 |
| factor(X25.CS)=1 | 1.17 | 21 | 1 | 0 | 0.2381 | 0.1624 | 0.3491 |
| factor(X25.CS)=1 | 1.38 | 20 | 1 | 0 | 0.2262 | 0.1523 | 0.3359 |
| factor(X25.CS)=1 | 1.42 | 19 | 3 | 0 | 0.1905 | 0.1226 | 0.2960 |
| factor(X25.CS)=1 | 2.26 | 16 | 1 | 0 | 0.1786 | 0.1129 | 0.2825 |
| factor(X25.CS)=1 | 2.72 | 15 | 3 | 0 | 0.1429 | 0.0846 | 0.2412 |
| factor(X25.CS)=1 | 3.39 | 12 | 2 | 0 | 0.1190 | 0.0665 | 0.2130 |
| factor(X25.CS)=1 | 3.68 | 10 | 0 | 1 | 0.1190 | 0.0665 | 0.2130 |
| factor(X25.CS)=1 | 4.61 | 9 | 0 | 1 | 0.1190 | 0.0665 | 0.2130 |
| factor(X25.CS)=1 | 4.87 | 8 | 0 | 2 | 0.1190 | 0.0665 | 0.2130 |
| factor(X25.CS)=1 | 5 | 6 | 0 | 6 | 0.1190 | 0.0665 | 0.2130 |

|  |  |  |  |  |  |  |  |  |  |
| --- | --- | --- | --- | --- | --- | --- | --- | --- | --- |
| X25.CS | records | n.max | n.start | events | \*rmean | \*se(rmean) | median | 0.95LCL | 0.95UCL |
| factor(X25.CS)=0 | 490 | 490 | 490 | 297 | 2.54 | 0.092 | 1.41 | 1.27 | 1.72 |
| factor(X25.CS)=1 | 84 | 84 | 84 | 74 | 1.143 | 0.173 | 0.29 | 0.23 | 0.69 |

Landmark analysis for time segment: EFS(years) > 5
Log rank test: implements the G-rho family of Harrington and Fleming (1982), with weights on each death of S(t)^rho, where S is the Kaplan-Meier estimate of survival. With rho = 0 this is the log-rank or Mantel-Haenszel test.

|  |  |  |  |
| --- | --- | --- | --- |
|  | N | Observed | Expected |
| factor(X25.CS)=0 | 95 | 1 | 0.9474 |
| factor(X25.CS)=1 | 6 | 0 | 0.0526 |

Chisq=
0.0556
on
1
degree of freedom, p=
0.8137
Survival table

|  |  |  |  |  |  |  |  |
| --- | --- | --- | --- | --- | --- | --- | --- |
|  | EFS(years) | N.Risk | N.Event | N.Censor | Survival | 95%CI Low | 95%CI Upp |
| factor(X25.CS)=0 | 5.02 | 95 | 0 | 1 | 1.0000 | 1.0000 | 1.0000 |
| factor(X25.CS)=0 | 5.04 | 94 | 0 | 1 | 1.0000 | 1.0000 | 1.0000 |
| factor(X25.CS)=0 | 5.17 | 93 | 0 | 1 | 1.0000 | 1.0000 | 1.0000 |
| factor(X25.CS)=0 | 5.18 | 92 | 0 | 1 | 1.0000 | 1.0000 | 1.0000 |
| factor(X25.CS)=0 | 5.2 | 91 | 0 | 2 | 1.0000 | 1.0000 | 1.0000 |
| factor(X25.CS)=0 | 5.27 | 89 | 0 | 2 | 1.0000 | 1.0000 | 1.0000 |
| factor(X25.CS)=0 | 5.3 | 87 | 0 | 1 | 1.0000 | 1.0000 | 1.0000 |
| factor(X25.CS)=0 | 5.33 | 86 | 0 | 1 | 1.0000 | 1.0000 | 1.0000 |
| factor(X25.CS)=0 | 5.35 | 85 | 0 | 1 | 1.0000 | 1.0000 | 1.0000 |
| factor(X25.CS)=0 | 5.36 | 84 | 0 | 1 | 1.0000 | 1.0000 | 1.0000 |
| factor(X25.CS)=0 | 5.37 | 83 | 0 | 2 | 1.0000 | 1.0000 | 1.0000 |
| factor(X25.CS)=0 | 5.38 | 81 | 0 | 1 | 1.0000 | 1.0000 | 1.0000 |
| factor(X25.CS)=0 | 5.41 | 80 | 0 | 1 | 1.0000 | 1.0000 | 1.0000 |
| factor(X25.CS)=0 | 5.44 | 79 | 0 | 2 | 1.0000 | 1.0000 | 1.0000 |
| factor(X25.CS)=0 | 5.46 | 77 | 0 | 1 | 1.0000 | 1.0000 | 1.0000 |
| factor(X25.CS)=0 | 5.52 | 76 | 0 | 1 | 1.0000 | 1.0000 | 1.0000 |
| factor(X25.CS)=0 | 5.54 | 75 | 0 | 2 | 1.0000 | 1.0000 | 1.0000 |
| factor(X25.CS)=0 | 5.57 | 73 | 0 | 1 | 1.0000 | 1.0000 | 1.0000 |
| factor(X25.CS)=0 | 5.58 | 72 | 1 | 0 | 0.9861 | 0.9594 | 1.0000 |
| factor(X25.CS)=0 | 5.6 | 71 | 0 | 1 | 0.9861 | 0.9594 | 1.0000 |
| factor(X25.CS)=0 | 5.61 | 70 | 0 | 1 | 0.9861 | 0.9594 | 1.0000 |
| factor(X25.CS)=0 | 5.63 | 69 | 0 | 1 | 0.9861 | 0.9594 | 1.0000 |
| factor(X25.CS)=0 | 5.67 | 68 | 0 | 1 | 0.9861 | 0.9594 | 1.0000 |
| factor(X25.CS)=0 | 5.7 | 67 | 0 | 1 | 0.9861 | 0.9594 | 1.0000 |
| factor(X25.CS)=0 | 5.75 | 66 | 0 | 1 | 0.9861 | 0.9594 | 1.0000 |
| factor(X25.CS)=0 | 5.79 | 65 | 0 | 1 | 0.9861 | 0.9594 | 1.0000 |
| factor(X25.CS)=0 | 5.81 | 64 | 0 | 2 | 0.9861 | 0.9594 | 1.0000 |
| factor(X25.CS)=0 | 5.84 | 62 | 0 | 1 | 0.9861 | 0.9594 | 1.0000 |
| factor(X25.CS)=0 | 5.92 | 61 | 0 | 1 | 0.9861 | 0.9594 | 1.0000 |
| factor(X25.CS)=0 | 5.95 | 60 | 0 | 1 | 0.9861 | 0.9594 | 1.0000 |
| factor(X25.CS)=0 | 6 | 59 | 0 | 1 | 0.9861 | 0.9594 | 1.0000 |
| factor(X25.CS)=0 | 6.04 | 58 | 0 | 1 | 0.9861 | 0.9594 | 1.0000 |
| factor(X25.CS)=0 | 6.12 | 57 | 0 | 1 | 0.9861 | 0.9594 | 1.0000 |
| factor(X25.CS)=0 | 6.16 | 56 | 0 | 1 | 0.9861 | 0.9594 | 1.0000 |
| factor(X25.CS)=0 | 6.17 | 55 | 0 | 1 | 0.9861 | 0.9594 | 1.0000 |
| factor(X25.CS)=0 | 6.35 | 54 | 0 | 1 | 0.9861 | 0.9594 | 1.0000 |
| factor(X25.CS)=0 | 6.37 | 53 | 0 | 1 | 0.9861 | 0.9594 | 1.0000 |
| factor(X25.CS)=0 | 6.4 | 52 | 0 | 1 | 0.9861 | 0.9594 | 1.0000 |
| factor(X25.CS)=0 | 6.45 | 51 | 0 | 1 | 0.9861 | 0.9594 | 1.0000 |
| factor(X25.CS)=0 | 6.46 | 50 | 0 | 1 | 0.9861 | 0.9594 | 1.0000 |
| factor(X25.CS)=0 | 6.48 | 49 | 0 | 1 | 0.9861 | 0.9594 | 1.0000 |
| factor(X25.CS)=0 | 6.49 | 48 | 0 | 1 | 0.9861 | 0.9594 | 1.0000 |
| factor(X25.CS)=0 | 6.5 | 47 | 0 | 2 | 0.9861 | 0.9594 | 1.0000 |
| factor(X25.CS)=0 | 6.51 | 45 | 0 | 2 | 0.9861 | 0.9594 | 1.0000 |
| factor(X25.CS)=0 | 6.52 | 43 | 0 | 1 | 0.9861 | 0.9594 | 1.0000 |
| factor(X25.CS)=0 | 6.53 | 42 | 0 | 1 | 0.9861 | 0.9594 | 1.0000 |
| factor(X25.CS)=0 | 6.57 | 41 | 0 | 1 | 0.9861 | 0.9594 | 1.0000 |
| factor(X25.CS)=0 | 6.58 | 40 | 0 | 2 | 0.9861 | 0.9594 | 1.0000 |
| factor(X25.CS)=0 | 6.63 | 38 | 0 | 1 | 0.9861 | 0.9594 | 1.0000 |
| factor(X25.CS)=0 | 6.64 | 37 | 0 | 1 | 0.9861 | 0.9594 | 1.0000 |
| factor(X25.CS)=0 | 6.79 | 36 | 0 | 1 | 0.9861 | 0.9594 | 1.0000 |
| factor(X25.CS)=0 | 6.81 | 35 | 0 | 1 | 0.9861 | 0.9594 | 1.0000 |
| factor(X25.CS)=0 | 6.96 | 34 | 0 | 1 | 0.9861 | 0.9594 | 1.0000 |
| factor(X25.CS)=0 | 7 | 33 | 0 | 1 | 0.9861 | 0.9594 | 1.0000 |
| factor(X25.CS)=0 | 7.08 | 32 | 0 | 1 | 0.9861 | 0.9594 | 1.0000 |
| factor(X25.CS)=0 | 7.21 | 31 | 0 | 1 | 0.9861 | 0.9594 | 1.0000 |
| factor(X25.CS)=0 | 7.26 | 30 | 0 | 1 | 0.9861 | 0.9594 | 1.0000 |
| factor(X25.CS)=0 | 7.36 | 29 | 0 | 2 | 0.9861 | 0.9594 | 1.0000 |
| factor(X25.CS)=0 | 7.38 | 27 | 0 | 1 | 0.9861 | 0.9594 | 1.0000 |
| factor(X25.CS)=0 | 7.41 | 26 | 0 | 1 | 0.9861 | 0.9594 | 1.0000 |
| factor(X25.CS)=0 | 7.45 | 25 | 0 | 1 | 0.9861 | 0.9594 | 1.0000 |
| factor(X25.CS)=0 | 7.48 | 24 | 0 | 1 | 0.9861 | 0.9594 | 1.0000 |
| factor(X25.CS)=0 | 7.54 | 23 | 0 | 1 | 0.9861 | 0.9594 | 1.0000 |
| factor(X25.CS)=0 | 7.55 | 22 | 0 | 1 | 0.9861 | 0.9594 | 1.0000 |
| factor(X25.CS)=0 | 7.56 | 21 | 0 | 2 | 0.9861 | 0.9594 | 1.0000 |
| factor(X25.CS)=0 | 7.62 | 19 | 0 | 1 | 0.9861 | 0.9594 | 1.0000 |
| factor(X25.CS)=0 | 7.68 | 18 | 0 | 1 | 0.9861 | 0.9594 | 1.0000 |
| factor(X25.CS)=0 | 7.74 | 17 | 0 | 3 | 0.9861 | 0.9594 | 1.0000 |
| factor(X25.CS)=0 | 7.94 | 14 | 0 | 2 | 0.9861 | 0.9594 | 1.0000 |
| factor(X25.CS)=0 | 7.98 | 12 | 0 | 1 | 0.9861 | 0.9594 | 1.0000 |
| factor(X25.CS)=0 | 8.01 | 11 | 0 | 1 | 0.9861 | 0.9594 | 1.0000 |
| factor(X25.CS)=0 | 8.17 | 10 | 0 | 1 | 0.9861 | 0.9594 | 1.0000 |
| factor(X25.CS)=0 | 8.21 | 9 | 0 | 1 | 0.9861 | 0.9594 | 1.0000 |
| factor(X25.CS)=0 | 8.27 | 8 | 0 | 1 | 0.9861 | 0.9594 | 1.0000 |
| factor(X25.CS)=0 | 8.36 | 7 | 0 | 1 | 0.9861 | 0.9594 | 1.0000 |
| factor(X25.CS)=0 | 8.49 | 6 | 0 | 2 | 0.9861 | 0.9594 | 1.0000 |
| factor(X25.CS)=0 | 8.72 | 4 | 0 | 1 | 0.9861 | 0.9594 | 1.0000 |
| factor(X25.CS)=0 | 8.84 | 3 | 0 | 1 | 0.9861 | 0.9594 | 1.0000 |
| factor(X25.CS)=0 | 9.26 | 2 | 0 | 1 | 0.9861 | 0.9594 | 1.0000 |
| factor(X25.CS)=0 | 9.95 | 1 | 0 | 1 | 0.9861 | 0.9594 | 1.0000 |
| factor(X25.CS)=1 | 5.33 | 6 | 0 | 1 | 1.0000 | 1.0000 | 1.0000 |
| factor(X25.CS)=1 | 5.38 | 5 | 0 | 1 | 1.0000 | 1.0000 | 1.0000 |
| factor(X25.CS)=1 | 6.4 | 4 | 0 | 1 | 1.0000 | 1.0000 | 1.0000 |
| factor(X25.CS)=1 | 7.12 | 3 | 0 | 1 | 1.0000 | 1.0000 | 1.0000 |
| factor(X25.CS)=1 | 7.46 | 2 | 0 | 1 | 1.0000 | 1.0000 | 1.0000 |
| factor(X25.CS)=1 | 8.1 | 1 | 0 | 1 | 1.0000 | 1.0000 | 1.0000 |

|  |  |  |  |  |  |  |  |  |  |
| --- | --- | --- | --- | --- | --- | --- | --- | --- | --- |
| X25.CS | records | n.max | n.start | events | \*rmean | \*se(rmean) | median | 0.95LCL | 0.95UCL |
| factor(X25.CS)=0 | 95 | 95 | 95 | 1 | 8.977 | 0.048 | NA | NA | NA |
| factor(X25.CS)=1 | 6 | 6 | 6 | 0 | 9.025 | 0 | NA | NA | NA |

Use subset of data: (!is.na(X5) & (X5==2))
Created by EmpowerStats (www.empowerstats.com) and R on 2025-10-07
